# Analysis of the trajectory of depressive symptoms and influencing factors in patients undergoing metabolic and bariatric surgery

**DOI:** 10.3389/fendo.2026.1789476

**Published:** 2026-06-10

**Authors:** Jia-li Zhang, Yi Zhu, Yan Shao, Jing Wang, Cheng-yan Pu, Xiao-yi Yang, Ya-jing Xu, Yi Shen

**Affiliations:** 1 Department of Gastroenterology Centre, The Affiliated Changzhou Second People’s Hospital of Nanjing Medical University, Changzhou, China; 2 Changzhou Medical Center, Nanjing Medical University, Changzhou, China; 3 Department of Cardiology, The Affiliated Changzhou Second People’s Hospital of Nanjing Medical University, Changzhou, China; 4 Department of Surgery, The Affiliated Changzhou Second People’s Hospital of Nanjing Medical University, Changzhou, China

**Keywords:** anxiety, depression, heterogeneous subgroups, MBS, obese

## Abstract

**Background and aims:**

This study aims to investigate the factors influencing depressive symptoms both before and after metabolic and bariatric surgery (MBS) in obese patients, as well as to analyze the contributing factors to heterogeneous subgroups with distinct depression trajectories.

**Methods:**

This study enrolled 184 obese patients who underwent MBS at Changzhou Second People’s Hospital between October 2023 and October 2024. Body composition and clinical laboratory data were obtained preoperatively, as well as at 1 month, 3 months, and 6 months postoperatively. The 9-item patient health questionnaire was used to assess the severity of depressive symptoms.

**Results:**

Depressive scores showed a significant decrease at 1 month, 3 months, and 6 months postoperatively. Multiple linear regression indicated that anxiety consistently served as a risk factor for the development of depression in MBS patients, while higher medication adherence, vitality, role emotional, and resting potential scores acted as protective factors against depression progression. Growth mixture model analysis identified two heterogeneous subgroups in the depression trajectories of MBS patients. Binary logistic regression revealed that patients with comorbid hypertension and low health transition scores exhibited lower preoperative depression levels and were more likely to belong to the “mild depression decline group”. In contrast, patients with comorbid anxiety, low medication adherence, and high vitamin B12 levels showed higher preoperative depression levels and tended to belong to the “significant depression decline group”.

**Conclusions:**

Patients with anxiety disorder are prone to developing depression after MBS. Patients with comorbid anxiety, low medication adherence, and high vitamin B12 levels exhibit higher preoperative depression severity and require particular attention. However, their postoperative depression levels decreased significantly, highlighting the importance of postoperative follow-up for effective emotional management in this subgroup.

## Introduction

Obesity is a chronic metabolic disease caused by the interaction of multiple factors such as dietary habits, socio-cultural background, neuropsychiatric factors, and endocrine imbalances (1). It has gradually become a public health issue that endangers residents’ health and increases the economic burden.

For obese patients with a body mass index (BMI) exceeding 32.5, metabolic and bariatric surgery (MBS) has become the preferred method for weight loss (2). MBS not only leads to long-term sustained weight reduction and improves health-related quality of life but also alleviates or even cures obesity-related metabolic complications such as hypertension, diabetes, hyperlipidemia, fatty liver disease, hyperuricemia, polycystic ovary syndrome, and sleep apnea syndrome (3). Obese patients often suffer not only from physical pathological and organic changes but also from negative emotions such as depression and anxiety. MBS, in turn, may increase the occurrence and severity of these negative emotions, which can further affect the effectiveness and sustainability of weight loss (4, 5). Therefore, early identification and timely intervention for negative emotions in MBS patients are particularly important.

Negative emotions primarily manifest as anxiety and depression, accompanied by psychological and physical symptoms such as tension, low mood, slowed reactions, sleep disorders, eating disorders, fatigue, and sexual dysfunction (6, 7). After MBS, patients may experience a series of postoperative complications, such as micronutrient deficiencies, gastric stenosis, and gastrointestinal reactions, which are typically more pronounced within 3 to 6 months after surgery (8). These complications may exacerbate the severity of depressive symptoms, thereby impacting weight loss outcomes (9). Currently, there is insufficient research on the dynamic changes in depressive emotions within 3 to 6 months after MBS, leading to a lack of evidence-based support for postoperative emotional management in these patients. Studies by Federico et al. (10) suggest that depressive emotions may not only reduce compliance and increase dropout rates among MBS patients but may also hinder their ability to maintain healthy eating and lifestyle habits, thereby limiting weight loss outcomes. Therefore, it is crucial to conduct perioperative and postoperative follow-ups on depressive emotions in MBS patients and analyze their influencing factors.

This study aims to investigate the current status of perioperative depressive emotions in MBS patients, understand the dynamic trajectory of depressive emotions, and analyze the influencing factors at different time points. The goal is to enable early detection of depressive emotions in patients undergoing metabolic and bariatric surgery and provide a theoretical basis for implementing early prevention and personalized management strategies.

## Patients and methods

### Ethics statement

This study was conducted in compliance with the Declaration of Helsinki and approved by the Committee of Clinical Investigation of the Affiliated Changzhou Second People’s Hospital of Nanjing Medical University ([2023]KY324-01). Written informed consent was obtained from all participants included in the study.

### Participants

This study is a prospective longitudinal observational study. A total of 200 obese patients who underwent metabolic and bariatric surgery in the Department of Metabolic and Bariatric Surgery at the Third Affiliated Hospital of Nanjing Medical University between October 2023 and October 2024 were selected. Inclusion criteria: (1) Undergoing laparoscopic sleeve gastrectomy for obesity for the first time; (2) BMI ≥ 30 kg/m² (WHO criteria); (3) Age ≥ 18 years; (4) Proficient in using a smartphone; (5) Educational level of primary school or higher. Exclusion criteria: (1) History of psychiatric disorders; (2) Currently receiving psychological therapy or taking psychotropic medications.

The general information questionnaire was self-designed by the researchers after reviewing relevant literature and was used to collect patient demographics, including age, gender, educational level, waist circumference, hip circumference, preoperative obesity-related comorbidities, personal history, and family history.

Waist circumference: The subject stands upright, with abdominal muscles relaxed. Measurement is taken at the end of a normal expiration at the midpoint between the lower border of the 12th rib and the top of the iliac crest (11).

Hip circumference: The subject stands upright, breathing normally, with arms hanging naturally at the sides, buttocks relaxed, and feet together. Measurement is taken horizontally at the level of the maximum prominence of the buttocks, with the tape measure passing anteriorly at the level of the pubic symphysis and posteriorly at the level of the greater trochanters (11).

Preoperative obesity-related comorbidities included type 2 diabetes, hypertension, hyperlipidemia, and sleep apnea syndrome. Personal history included smoking history and alcohol consumption history. Family history included familial obesity and history of psychiatric disorders.

**Figure 1:**
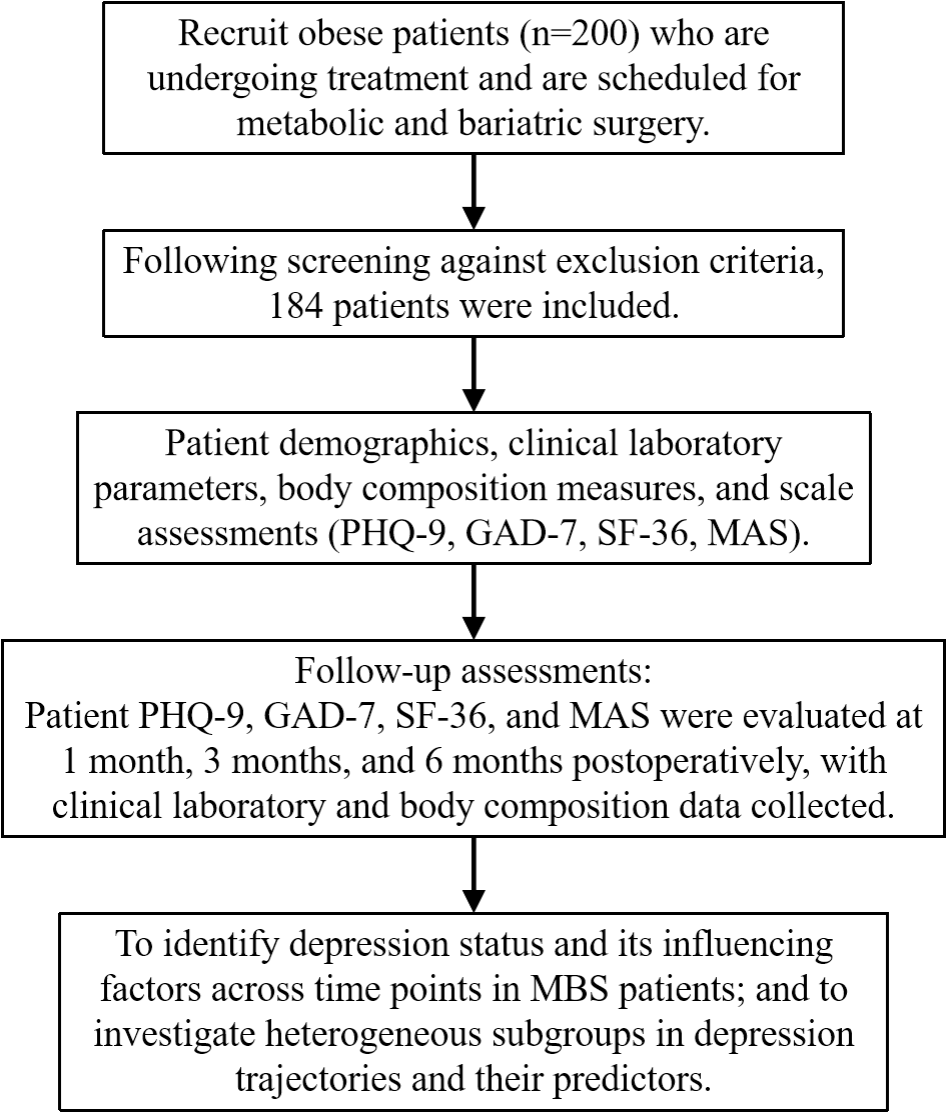
Flow chart.

The general information questionnaire was used to collect baseline demographic data (e.g., age, gender, smoking history, alcohol consumption history, and medical history) from patients prior to MBS. Assessments using the 9-Item Patient Health Questionnaire (PHQ-9), the 7-Item Generalized Anxiety Disorder Scale (GAD-7), the 36-Item Short Form Health Survey (SF-36), and the Medication Adherence Scale (MAS) scales were conducted at the following time points: preoperatively (T0), and at 1 month (T1), 3 months (T2), and 6 months (T3) postoperatively. The scales were completed on-site by the patients. To maximize the accuracy of the responses, the researcher provided a private, quiet, and independent space for this purpose. The researcher also patiently addressed any questions or clarifications the patients had regarding the scale items. Concurrently, body composition indicators and laboratory test results were collected from the MBS patients at each of the T0 to T3 time points (**Figure 1** Flow chart). In this study, the staff personally collected blood samples from the patients and performed anthropometric measurements.

### The 9-item patient health questionnaire

This scale is a self-assessment tool specifically designed for the evaluation of mental disorders in primary care. Known for its simplicity and efficiency, it is widely used for screening mental disorders in primary healthcare settings. A PHQ-9 score of ≥10 has a sensitivity of 88% and a specificity of 88%, with a positive predictive value ranging from 31% to 51%. The questionnaire consists of 9 items, each rated on a 4-point scale from 0 to 3. The total score is interpreted as follows: 5–9 indicates mild depressive symptoms, 10–14 indicates moderate depressive symptoms, 15–19 indicates moderately severe depressive symptoms, and 20–27 indicates severe depressive symptoms (12, 13).

### The 7-item generalized anxiety disorder scale

This scale is a simple and effective assessment tool for screening generalized anxiety disorder. A GAD-7 score of ≥10 has a sensitivity of 86.2% and a specificity of 95.5%. The questionnaire consists of 7 items, each rated on a 4-point scale from 0 to 3. The total score is interpreted as follows: 5–9 indicates mild anxiety, 10–14 indicates moderate anxiety, and 15–21 indicates severe anxiety (14).

### The 36-item short form health survey

The SF-36 questionnaire consists of 9 dimensions, including Physical Functioning (PF), Role-Physical (RP), Bodily Pain (BP), General Health (GH), Vitality (VT), Social Functioning (SF), Role-Emotional (RE), Mental Health (MH), and Health Transition (HT). These can be further aggregated into a Physical Component Summary (including PF, RP, BP, GH) and a Mental Component Summary (including VT, SF, RE, MH). Since the number of items varies across dimensions, the raw scores obtained from respondents’ self-assessments are recalibrated. The converted score for each dimension = [(Actual Score - Lowest Possible Score)/Possible Score Range] × 100. The total score of the SF-36 scale is the average of the sum of the 9 dimension scores. A higher score indicates a better health-related quality of life, while a lower score indicates a poorer health-related quality of life (15, 16).

### The medication adherence scale

The MAS is widely used to assess medication adherence in patients with chronic conditions. The scale encompasses medication compliance and psychosocial factors related to medication, characterized by its comprehensiveness and high specificity. It consists of 4 dimensions and 12 items, as follows: Medication Adherence (Items 1-3), Collaboration with Healthcare Providers on Medication Use (Items 4-6), Willingness to Acquire and Use Medication Knowledge (Items 7-9), and Willingness to Integrate Medication into Daily Life (Items 10-12). The total score ranges from 12 to 60, with a higher score indicating better medication adherence (17).

### Statistical analyses

Statistical analysis was performed using SPSS 28.0 and R studio. Normally distributed continuous data were described as mean ± standard deviation, while non-normally distributed continuous data were described as median (25%-75% interquartile range). Categorical data were described using frequencies. For the quantitative data measured across the four time points: if the data met the assumptions of normality and sphericity, repeated measures analysis of variance (ANOVA) was employed. Sphericity was tested using Mauchly’s test; if the p-value was > 0.05, the sphericity assumption was met. If the p-value was < 0.05, indicating a violation of sphericity, the degrees of freedom were adjusted using the Greenhouse-Geisser correction. If the data were non-normally distributed, non-parametric rank-sum tests were used. Pearson or Spearman correlation tests were used to examine the correlations between variables at time points T0 to T3 and the PHQ-9 scores. Multiple linear regression analysis was applied to identify factors influencing the PHQ-9 scores at the T0 to T3 time points. A Growth Mixture Model (GMM) was used to explore population heterogeneity in the developmental trajectories of depression among MBS patients from T0 to T3. Chi-square tests and independent samples t-tests were used to compare differences in general demographics, scale scores, body composition, and clinical laboratory indicators across the identified different depression trajectory groups. Binary logistic regression and multiple linear regression analyses were employed to examine the influence of general demographics, scale scores, body composition, and clinical laboratory indicators on the heterogeneous subgroups of depression trajectories in MBS patients. A two-tailed p-value of < 0.05 was considered statistically significant.

## Results

A total of 184 patients were included in the study. The mean age of the participants was 32.6 ± 7.5 years, with a maximum age of 53 years. Among them, 55 (29.9%) were male and 129 (70.1%) were female. Males constituted 30% of the total patient population, and the mean age was only 32.6 years, which is generally consistent with the mean age of the male population undergoing MBS nationwide. Smokers accounted for 14.7%, and alcohol consumers accounted for 13.6% of the cohort. Additionally, 27.7% of patients had hypertension, 18.5% had type 2 diabetes, and 5.4% had sleep apnea syndrome. The results of the repeated measures analysis of variance indicated that the body composition indicators (BMI, waist circumference, hip circumference, mid-upper arm circumference, thigh circumference, body fat percentage, basal metabolic rate) and clinical laboratory parameters (HbA1c, ALT, AST, total cholesterol, HDL-C, LDL-C) in MBS patients gradually decreased over time. In contrast, clinical laboratory parameters (Vitamin D, folic acid) showed a gradual increase over time. The differences across the various time points were statistically significant, suggesting a general improvement in body composition and most clinical laboratory parameters (except for vitamin B12) within the first 6 months after surgery. At the 6-month postoperative mark, patients’ depression scores showed a decreasing trend over time, indicating a gradual alleviation of depressive symptoms. The repeated measures ANOVA results confirmed that the differences in depression scores across the four time points were statistically significant. Furthermore, the GAD-7 scores of MBS patients also decreased significantly over time, suggesting that anxiety symptoms gradually improved during the 6-month postoperative period. Scores on the MAS and SF-36 (including its subdomains) generally exhibited an increasing trend over time, with statistically significant differences across time points, indicating an overall improvement in medication adherence and quality of life (**Table 1**).

**Table 1:** Profiles of clinical laboratory parameters, body composition, and scale scores in MBS patients at the four follow-up time points.

Characteristics (n = 184)	T0	T1	T2	T3	P value
Age (years)	32.6 ± 7.5				
Sex, male, n (%)	55 (29.9)				
Smoking, n (%)	27 (14.7)				
Alcohol consumption, n (%)	25 (13.6)				
Hypertension, n (%)	51 (27.7)				
Diabetes, n (%)	34 (18.5)				
OSASH, n (%)	10 (5.4)				
Biochemical test					
ALT (U/L)	56.9 ± 34.9	30.9 ± 21.9	33.3 ± 27.5	27.8 ± 22.1	< 0.001
AST (U/L)	39.9 ± 19.9	25.2 ± 12.3	26.8 ± 17.6	23.0 ± 13.7	< 0.001
LDL-C (mmol/L)	3.0 ± 0.8	2.9 ± 0.7	2.8 ± 0.8	2.6 ± 0.7	< 0.001
HDL-C (mmol/L)	1.1 ± 0.2	1.0 ± 0.2	1.1 ± 0.2	1.1 ± 0.3	0.007
TC (mmol/L)	4.6 ± 0.9	4.4 ± 0.9	4.5 ± 1.0	4.1 ± 0.9	< 0.001
Vit D (nmol/L)	62.0 ± 22.4	69.6 ± 22.6	77.2 ± 22.7	84.6 ± 22.9	< 0.001
Vit B12 (pg/ml)	578.9 ± 211.8	579.5 ± 215.5	579.4 ± 218.1	578.7 ± 223.2	0.957
Folic acid (ng/mL)	11.5 ± 4.9	12.0 ± 4.9	12.5 ± 4.9	13.1 ± 5.1	< 0.001
HbA1c (%)	6.0 ± 1.2	5.7 ± 1.0	5.5 ± 0.7	5.4 ± 0.6	< 0.001
Body composition indicators					
BMI (kg/m2)	37.9 ± 5.9	33.6 ± 5.8	31.7 ± 5.3	27.8 ± 4.4	< 0.001
Waist circumference (cm)	120.1 ± 14.3	110.1 ± 14.7	102.3 ± 12.5	89.5 ± 10.8	< 0.001
Hip circumference (cm)	121.2 ± 11.4	114.3 ± 10.9	104.7 ± 9.6	91.3 ± 9.7	< 0.001
Mid-upper arm Circumference (cm)	35.6 ± 4.1	33.3 ± 5.6	30.7 ± 4.1	26.6 ± 3.1	< 0.001
Thigh circumference (cm)	67.9 ± 7.2	63.7 ± 6.5	58.6 ± 6.4	50.9 ± 6.1	< 0.001
Body fat percentage (%)	44.5 ± 5.8	42.2 ± 6.5	37.6 ± 5.7	32.6 ± 4.7	< 0.001
Basal metabolic rate (kcal)	1622 ± 233	1529 ± 229	1428 ± 208	1240 ± 207	< 0.001
Scale assessment					
PHQ-9 (points)	6.4 ± 5.3	5.8 ± 5.1	4.2 ± 3.8	2.4 ± 3.3	< 0.001
GAD-7 (points)	4.4 ± 4.1	4.2 ± 4.1	2.8 ± 3.3	1.8 ± 2.4	< 0.001
MAS (points)	40.3 ± 11.6	46.8 ± 9.1	49.2 ± 7.9	48.7 ± 6.5	< 0.001
SF-36 (points)	50.2 ± 9.1	50.8 ± 6.4	56.4 ± 4.7	63.3 ± 5.0	< 0.001
PF (points)	57.5 ± 20.8	59.2 ± 21.7	65.3 ± 21.5	77.0 ± 19.6	< 0.001
RP (points)	53.8 ± 30.9	53.6 ± 33.0	66.4 ± 28.4	79.1 ± 25.8	< 0.001
BP (points)	29.9 ± 17.2	28.7 ± 16.4	27.7 ± 16.2	32.5 ± 14.1	0.001
GH (points)	57.7 ± 10.7	55.7 ± 11.6	56.8 ± 9.9	60.5 ± 10.2	< 0.001
VT (points)	57.6 ± 14.4	59.7 ± 11.9	61.0 ± 13.0	65.1 ± 9.1	< 0.001
SF (points)	41.2 ± 15.6	46.3 ± 13.9	48.7 ± 15.3	49.7 ± 12.6	< 0.001
RE (points)	51.4 ± 39.5	70.1 ± 32.4	84.6 ± 23.3	90.9 ± 20.1	< 0.001
MH (points)	52.2 ± 13.6	51.5 ± 12.6	54.7 ± 13.1	57.6 ± 11.2	< 0.001
HT (points)	50.7 ± 29.0	32.3 ± 22.6	42.4 ± 28.2	57.6 ± 26.3	< 0.001

Values are shown as the means ± SD, median (interquartile range) or percentage. Assessment Time Points: T0 (preoperative), T1 (1-month postoperative), T2 (3-months postoperative), T3 (6-months postoperative). OSASH obstructive sleep apnea syndrome with hypoxemia, ALT alanine aminotransferase, AST aspartate aminotransferase, LDL-C low-density lipoprotein cholesterol, HDL-C high-density lipoprotein cholesterol, TC total cholesterol, BMI body mass index, PHQ-9 the 9-item patient health questionnaire, GAD-7 the 7-item generalized anxiety disorder scale, MAS the medication adherence scale, SF-36 the 36-item short form health survey, consists of 9 dimensions, including Physical Functioning (PF), Role-Physical (RP), Bodily Pain (BP), General Health (GH), Vitality (VT), Social Functioning (SF), Role-Emotional (RE), Mental Health (MH), and Health Transition (HT).

A Pearson correlation analysis was conducted to examine the relationships between PHQ-9 scores and body composition indicators, as well as clinical laboratory parameters, in MBS patients across time points T0 to T3 (**Table 2**). The results revealed that at T0 (preoperative), patients’ PHQ-9 scores showed a positive correlation with body fat percentage. At T1 (1-month postoperative), a positive correlation was observed between PHQ-9 scores and HDL-C levels. However, no significant correlations were found between PHQ-9 scores and any of the body composition or clinical laboratory indicators at the T2 and T3 time points. Furthermore, Pearson correlation analysis was performed between PHQ-9 scores and GAD-7 scores, MAS scores, SF-36 total scores, and its subdomain scores across the four time points. The results indicated: At T0, PHQ-9 scores were positively correlated with GAD-7 and BP scores, while negatively correlated with MAS scores, SF-36 total score, and the PF, RP, VT, and RE subdomain scores. At T1, PHQ-9 scores were positively correlated with GAD-7 scores and negatively correlated with MAS scores, SF-36 total score, and the SF subdomain score. At T2, PHQ-9 scores were positively correlated with GAD-7, BP, and GH scores, and negatively correlated with MAS scores and the SF-36 total score. At T3, PHQ-9 scores were positively correlated with GAD-7 and BP scores, and negatively correlated with the SF-36 total score, RP, GH, SF, and RE subdomain scores.

**Table 2:** Correlations of factors with depression in MBS patients over time (T0 to T3).

Characteristics	T0		T1		T2		T3	
	r	P value	r	P value	r	P value	r	P value
ALT	0.017	0.824	0.017	0.824	-0.031	0.674	0.008	0.918
AST	0.042	0.569	0.042	0.569	0.009	0.902	-0.017	0.815
LDL-C	-0.025	0.734	0.032	0.664	0.036	0.634	0.089	0.235
HDL-C	-0.083	0.26	0.155	0.036	0.018	0.807	0.047	0.527
TC	0.128	0.082	0.071	0.34	0.061	0.417	0.111	0.137
Vit D	0.012	0.874	0.03	0.69	-0.016	0.827	-0.103	0.169
Vit B12	0.103	0.162	0.108	0.145	0.031	0.683	0.064	0.394
Folic acid	0.028	0.704	0.075	0.311	0.059	0.428	-0.01	0.89
HbA1c	0.072	0.332	-0.132	0.074	-0.058	0.434	-0.05	0.502
BMI	0.023	0.753	0.008	0.915	0.061	0.412	-0.055	0.463
Waist circumference	0.082	0.268	0.015	0.842	-0.002	0.979	0.042	0.578
Hip circumference	0.004	0.955	-0.013	0.859	-0.015	0.838	0.008	0.919
Mid-upper arm circumference	0.002	0.976	-0.033	0.656	-0.053	0.48	0.022	0.77
Thigh circumference	-0.075	0.313	-0.079	0.288	-0.085	0.252	-0.029	0.9
Body fat percentage	0.161	0.029	0.05	0.501	0.085	0.253	0.038	0.612
Basal metabolic rate	-0.05	0.502	-0.021	0.779	-0.138	0.063	-0.071	0.343
GAD-7	0.815	< 0.001	0.836	< 0.001	0.836	< 0.001	0.866	< 0.001
MAS	-0.835	< 0.001	-0.401	< 0.001	-0.401	< 0.001	-0.108	0.147
SF-36	-0.302	< 0.001	-0.192	< 0.001	-0.192	< 0.001	-0.311	< 0.001
PF	-0.239	0.001	-0.095	0.201	-0.105	0.157	-0.119	0.112
RP	-0.24	0.001	0.032	0.664	-0.064	0.388	-0.153	0.04
BP	0.15	0.042	0.054	0.468	0.209	0.004	0.173	0.02
GH	0.09	0.225	0.031	0.679	0.153	0.038	-0.146	0.04
VT	-0.221	0.003	0.017	0.822	-0.022	0.765	-0.085	0.255
SF	-0.047	0.523	-0.187	0.011	-0.07	0.347	-0.178	0.016
RE	-0.314	< 0.001	-0.138	0.063	-0.103	0.163	-0.319	< 0.001
MH	-0.11	0.135	-0.109	0.139	-0.087	0.242	0.002	0.981
HT	0.065	0.381	-0.039	0.595	-0.061	0.411	0.027	0.721

Assessment Time Points: T0 (preoperative), T1 (1-month postoperative), T2 (3-months postoperative), T3 (6-months postoperative). ALT alanine aminotransferase, AST aspartate aminotransferase, LDL-C low-density lipoprotein cholesterol, HDL-C high-density lipoprotein cholesterol, TC total cholesterol, BMI body mass index, PHQ-9 the 9-item patient health questionnaire, GAD-7 the 7-item generalized anxiety disorder scale, MAS the medication adherence scale, SF-36 the 36-item short form health survey, consists of 9 dimensions, including Physical Functioning (PF), Role-Physical (RP), Bodily Pain (BP), General Health (GH), Vitality (VT), Social Functioning (SF), Role-Emotional (RE), Mental Health (MH), and Health Transition (HT).

Multiple linear regression analyses were performed with the PHQ-9 scores of MBS patients at time points T0 through T3 as the dependent variables, respectively (**Table 3**). The independent variables included were those identified as statistically significant in the preceding correlation analyses. Collinearity diagnostics indicated that the tolerance for all independent variables was greater than 0.2 and the Variance Inflation Factor was less than 10, suggesting an absence of multicollinearity among the predictors. The results revealed the following statistically significant independent predictors of PHQ-9 scores at each time point: At T0, GAD-7 scores, VT scores, and RE scores; At T1: GAD-7 scores, MAS scores, and HDL-C levels; At T2: GAD-7 scores. At T3: GAD-7 scores, RP scores, and RE scores.

**Table 3:** Multiple linear regression analysis of factors influencing PHQ-9 scores at each time point.

Characteristics	B	SE	B’	t	95 % CI		P Value
					Upper	Lower	
T0							
GAD-7	0.9	0.065	0.703	13.796	0.771	1.028	< 0.001
MAS	-0.039	0.021	-0.086	-1.833	-0.081	0.003	0.069
SF-36	0.076	0.064	0.13	1.186	-0.05	0.201	0.237
BP	-0.024	0.019	-0.078	-1.273	-0.062	0.013	0.205
PF	-0.031	0.018	-0.123	-1.771	-0.066	0.004	0.078
RP	-0.008	0.011	-0.048	-0.752	-0.03	0.013	0.453
VT	-0.042	0.019	-0.115	-2.245	-0.079	-0.005	0.026
RE	-0.026	0.001	-0.196	-2.485	-0.047	-0.005	0.014
Body fat percentage	0.041	0.041	0.045	1.008	-0.04	0.122	0.315
T1							
GAD-7	0.976	0.054	0.792	18.22	0.845	1.103	< 0.001
MAS	-0.067	0.024	-0.119	-2.749	-0.125	-0.016	0.007
SF-36	-0.024	0.033	-0.03	-0.724	-0.089	0.037	0.47
SF	0.004	0.015	0.02	0.246	-0.021	0.028	0.806
HDL-C	2.32	0.91	0.101	2.551	0.601	3.901	0.012
T2							
GAD-7	0.894	0.053	0.778	16.728	0.726	1.034	< 0.001
MAS	0.027	0.022	0.056	1.225	-0.012	0.064	0.222
SF-36	-0.007	0.039	-0.009	-0.182	-0.095	0.07	0.856
BP	0.015	0.012	0.063	1.297	-0.008	0.039	0.196
GH	0.023	0.019	0.06	1.229	-0.013	0.062	0.221
T3							
GAD-7	1.142	0.054	0.836	21.037	0.888	1.315	< 0.001
SF-36	0.043	0.038	0.065	1.134	-0.052	0.133	0.258
BP	0.003	0.011	0.011	0.237	-0.019	0.026	0.813
RP	-0.012	0.006	-0.096	-2.084	-0.027	0.002	0.039

Assessment Time Points: T0 (preoperative), T1 (1-month postoperative), T2 (3-months postoperative), T3 (6-months postoperative). GAD-7 the 7-item generalized anxiety disorder scale, MAS the medication adherence scale, SF-36 the 36-item short form health survey, consists of 9 dimensions, including Physical Functioning (PF), Role-Physical (RP), Bodily Pain (BP), General Health (GH), Vitality (VT), Social Functioning (SF), Role-Emotional (RE), Mental Health (MH), and Health Transition (HT).

**Figure 2:**
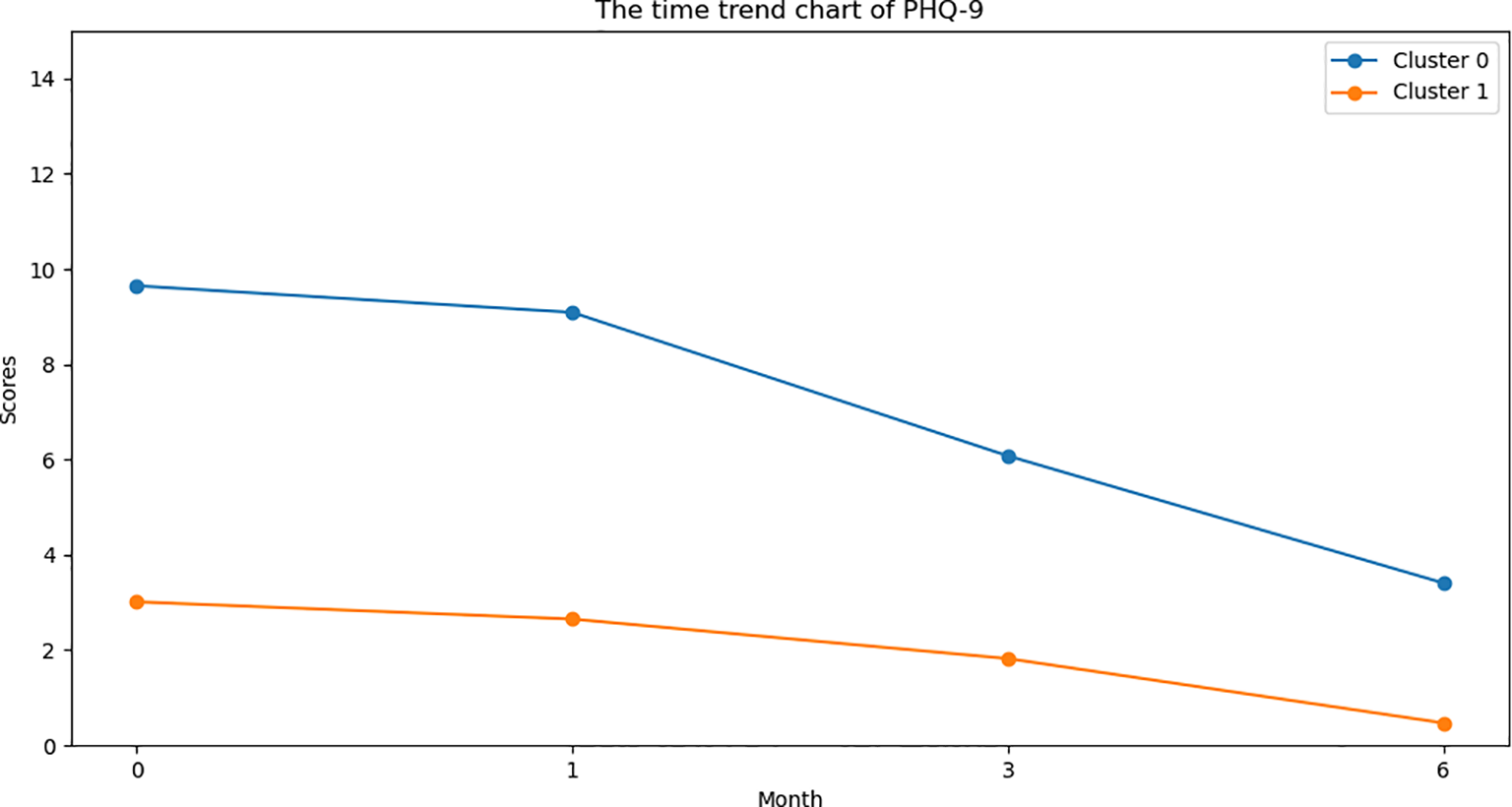
Trajectory of depressive mood in MBS patients during the 6-month postoperative period.

Using GMM, the trajectory of depressive mood changes in MBS patients was divided into two subgroups. Cluster 0 is the first subgroup, comprising 90 cases, accounting for 48.9% of the total. The depressive mood in this group generally exhibited a trend of “initially high depression levels, followed by a slow decline, and then a rapid decrease,” and can be named the “significant depression decline group.” Cluster 1 is the second subgroup, comprising 94 cases, accounting for 51.1% of the total. The depressive mood in this group generally showed characteristics of “initially low depression levels, followed by a slow decline, with minimal overall change and relative stability,” and can be named the “mild depression decline group.” The trends of depressive mood changes over time for the two subgroups are detailed in **Figure 2**. The quadratic functions fitted to the trajectory curves of the two subgroups are as follows: Cluster 0: 9.939 - 1.302 Time + 0.034 Time², Cluster 1: 3.030 - 0.377 Time - 0.0076 Time².

To explore the factors influencing the heterogeneous subgroups of depression trajectories in MBS patients, this study treated the two subgroups as the dependent variable, with baseline (T0) demographics, GAD-7, MAS, SF-36, body composition indicators, and clinical laboratory parameters as independent variables, and performed univariate analysis. As shown in **Table 4**, there were no statistically significant differences between the groups in terms of age, sex, smoking history, drinking history, presence of type 2 diabetes, or sleep apnea syndrome. The proportion of patients with hypertension was significantly higher in the mild depression decline group (Cluster 1) than in the significant depression decline group (Cluster 0). Additionally, statistically significant differences were observed between the significant depression decline group and the mild depression decline group in GAD-7 scores, MAS scores, VT scores, HT scores, body fat percentage, and vitamin B12 levels.

**Table 4:** Univariate analysis of depression trajectory subgroups in MBS patients.

Characteristics (n = 184)	Cluster 0 (n = 90)	Cluster 1 (n = 94)	P value
Age (years)	32.4 ± 7.9	32.8 ± 7.1	0.678
Sex, male, n (%)	24 (26.7)	31 (33.0)	0.350
Smoking, n (%)	17 (18.9)	10 (10.6)	0.114
Alcohol consumption, n (%)	15 (16.7)	10 (10.6)	0.233
Hypertension, n (%)	18 (20.0)	33 (35.1)	0.022
Diabetes, n (%)	12 (13.3)	22 (23.4)	0.079
OSASH, n (%)	2 (2.2)	8 (8.5)	0.060
Biochemical test			
ALT (U/L)	61.2 ± 38.7	52.6 ± 30.6	0.104
AST (U/L)	42.0 ± 20.1	37.8 ± 19.6	0.156
LDL-C (mmol/L)	2.97 ± 0.85	3.02 ± 0.77	0.677
HDL-C (mmol/L)	1.05 ± 0.23	1.07 ± 0.22	0.627
TC (mmol/L)	4.6 ± 1.0	4.6 ± 0.9	0.328
Vit D (nmol/L)	61.9 ± 22.7	62.1 ± 22.3	0.980
Vit B12 (pg/ml)	625 ± 210	534 ± 204	0.004
Folic acid (ng/mL)	12.0 ± 4.9	11.1 ± 4.9	0.182
HbA1c (%)	5.9 ± 1.2	6.1 ± 1.2	0.443
Body composition indicators			
BMI (kg/m2)	37.9 ± 6.2	37.7 ± 5.7	0.844
Waist circumference (cm)	119.6 ± 13.3	120.6 ± 15.2	0.651
Hip circumference (cm)	120.5 ± 11.0	121.7 ± 11.7	0.462
Mid-upper arm circumference (cm)	35.1 ± 3.6	36.1 ± 4.4	0.079
Thigh circumference (cm)	67.1 ± 6.9	68.6 ± 7.3	0.117
Body fat percentage (%)	45.4 ± 5.3	43.5 ± 5.9	0.028
Basal metabolic rate (kcal)	1583 ± 204	1626 ± 343	0.310
Scale assessment			
GAD-7 (points)	6.9 ± 4.2	2.0 ± 2.2	< 0.001
MAS (points)	35.9 ± 12.3	44.4 ± 9.3	< 0.001
SF-36 (points)	50.1 ± 9.6	50.4 ± 8.6	0.854
PF (points)	56.6 ± 20.2	58.3 ± 21.4	0.584
RP (points)	52.7 ± 32.8	54.7 ± 28.9	0.661
BP (points)	30.9 ± 18.4	28.9 ± 16.1	0.428
GH (points)	56.7 ± 10.7	58.7 ± 10.8	0.196
VT (points)	53.7 ± 14.8	61.2 ± 13.1	< 0.001
SF (points)	40.7 ± 17.3	41.6 ± 13.7	0.687
RE (points)	51.9 ± 42.3	51.1 ± 36.8	0.893
MH (points)	52.0 ± 14.2	52.4 ± 12.9	0.833
HT (points)	55.6 ± 28.9	46.0 ± 28.4	0.025

ALT alanine aminotransferase, AST aspartate aminotransferase, LDL-C low-density lipoprotein cholesterol, HDL-C high-density lipoprotein cholesterol, TC total cholesterol, BMI body mass index, PHQ-9 the 9-item patient health questionnaire, GAD-7 the 7-item generalized anxiety disorder scale, MAS the medication adherence scale, SF-36 the 36-item short form health survey, consists of 9 dimensions, including Physical Functioning (PF), Role-Physical (RP), Bodily Pain (BP), General Health (GH), Vitality (VT), Social Functioning (SF), Role-Emotional (RE), Mental Health (MH), and Health Transition (HT).

Binary logistic regression was performed with the significant depression decline group and the mild depression decline group as the dependent variable, and hypertension comorbidity, GAD-7 score, MAS score, VT score, HT score, body fat percentage, and vitamin B12 level as independent variables, to analyze influencing factors for heterogeneous trajectory subgroups in MBS patients. The results showed that the presence of hypertension, GAD-7 score, MAS score, HT score, and vitamin B12 level were significant factors influencing trajectory development toward the significant decline group. Specifically, the risk of belonging to the mild depression decline group was 2.666 times higher in patients with hypertension than in those without (OR = 2.666, 95% CI = 1.086–6.546, P = 0.032). The risk was 0.082 times higher in patients with higher anxiety levels compared to those with lower levels (OR = 0.082, 95% CI = 0.035–0.192, P < 0.001). Patients with lower medication adherence had a risk 0.444 times that of those with higher adherence (OR = 0.444, 95% CI = 0.204–0.966, P = 0.041). Patients with lower health transition scores had a risk 4.092 times higher than those with higher scores (OR = 4.092, 95% CI = 1.720–9.734, P = 0.001). Lastly, patients with lower vitamin B12 levels had a risk 0.190 times that of those with higher levels (OR = 0.190, 95% CI = 0.084–0.431, P < 0.001). For details, see **Table 5**.

**Table 5:** Binary logistic regression analysis of heterogeneous subgroups in depressive patients undergoing MBS.

Characteristics	B	OR	95 % CI	P value
Hypertension	0.981	2.666	1.086 - 6.546	0.032
GAD-7 > 3 points	-2.501	0.082	0.035 - 0.192	< 0.001
MAS < 43.5 points	-0.811	0.444	0.204 - 0.966	0.041
VT < 60 points	-0.534	0.586	0.269 - 1.278	0.179
HT < 50 points	1.409	4.092	1.720 - 9.734	0.001
Body Fat Percentage > 44.75 %	0.174	1.191	0.554 - 2.560	0.655
Vit B12 > 616.223 pg/ml	-1.662	0.19	0.084 - 0.431	< 0.001

GAD-7, the 7-item generalized anxiety disorder scale; MAS, the medication adherence scale; VT, Vitality; HT, Health Transition.

## Discussion

Among MBS patients, the most common type of mental disorder is depression (18). Perioperative depressive symptoms in MBS patients may increase postoperative pain, gastrointestinal discomfort, and reduced physical activity, thereby raising healthcare costs (19, 20). This study used a growth mixture model to fit the depression trajectory in MBS patients. 48.9% of MBS patients were classified into the “significant depression decline group.” These patients had high preoperative depression levels, which declined slowly until one month postoperatively, followed by a rapid decline from one to six months postoperatively. This suggests that such patients experience severe preoperative depression and significant psychological burden, but their mood may improve markedly as weight loss and metabolic indicators improve. For these patients, healthcare providers should adopt phased and targeted health management measures: from preoperative to one month postoperatively, the focus should be on providing psychological support and building confidence in weight loss; from one to six months postoperatively, attention should shift to social function recovery and quality of life improvement, facilitating a comprehensive return to normal life.

51.1% of MBS patients were classified into the “mild depression decline group.” These patients had low preoperative depression levels, which declined slowly over time with minimal overall change and remained relatively stable. These patients exhibited better psychological adaptability, but healthcare providers should remain vigilant about the potential impact of long-term mild depression on metabolic indicators and weight loss outcomes. Screening for “smiling depression” is also recommended. From preoperative to six months postoperatively, health management should focus on enhancing self-efficacy through peer education and personalized guidance.

This study found that comorbid hypertension is a risk factor for heterogeneous subgroups of depression trajectories in MBS patients. Patients with hypertension had a significantly higher risk of belonging to the “significant depression decline group.” Cardiovascular diseases and psychological disorders interact bidirectionally: patients with cardiovascular diseases are more prone to psychological issues, while psychological disorders can negatively influence the onset and progression of cardiovascular diseases (21). Additionally, patients with higher anxiety levels had 0.082 times the risk of belonging to the significant depression decline group compared to those with lower anxiety levels, indicating a close relationship between anxiety and depression progression. Higher anxiety levels may impede depression relief. Studies have shown a high comorbidity rate between anxiety and depression, with point prevalence rates of comorbid depression in anxiety disorder patients ranging from 2% to 69%, and lifetime prevalence as high as 81% (22). This high comorbidity may be linked to shared neurobiological mechanisms, such as abnormal brain network connectivity related to cb factors and genetic risks (22). Post-stroke patients also experience comorbid anxiety and depression, which reduces their quality of life (23). Telephone-based cognitive behavioral therapy has been shown to improve anxiety, depression, and quality of life in these patients (24). Other studies have found that internet-based acceptance and commitment therapy effectively reduces repetitive negative thinking in patients with comorbid anxiety and depression, thereby significantly alleviating depressive and anxiety symptoms (25, 26). These findings suggest that early psychological assessment and intervention, along with analysis of intervention effects and exploration of mechanisms underlying anxiety-depression comorbidity, are essential in clinical practice.

Patients with lower medication adherence had 0.444 times the risk of belonging to the significant depression decline group compared to those with higher adherence. Studies indicate that medication non-adherence affects 50% to 70% of patients with depressive disorders, influenced by factors such as illness experience, motivation, family and social support, and medication management plans (27). Non-adherence leads to fluctuations in drug concentration, impairing efficacy and delaying depression improvement. Health transition, one dimension of the SF-36 scale, was also significant in this study. Patients with lower scores had 4.092 times the risk of belonging to the significant depression decline group compared to those with higher scores. Previous studies have shown that health transition scores are positively correlated with depression levels in maintenance hemodialysis patients, elderly type 2 diabetes patients, and community-dwelling older adults (28, 29). Combined with the findings of this study, this suggests a close association between health perception and mental health. Nursing models based on the Information-Motivation-Behavioral Skills model have been proven to improve various dimensions of quality of life and alleviate depressive symptoms (30, 31). This finding underscores the importance of not only focusing on patients’ objective health status but also paying attention to their subjective health evaluations in clinical practice. Improving patients’ health perception may represent a new approach to preventing and alleviating depressive symptoms.

This study has several limitations: (1) The follow-up period was relatively short. A six-month follow-up is insufficient to explore the long-term depression trajectories of patients in the significant and mild decline groups after metabolic and bariatric surgery. Future studies could extend the follow-up duration to investigate long-term depression trajectories in obese patients post-surgery. (2) The study sample was limited to patients from a single center, which may restrict generalizability. Future research could involve multi-center studies to increase sample size and improve representativeness. (3) Survey results may be subject to subjective bias from the included patients. Future studies could incorporate objective indicators and employ more rigorous randomized controlled trials to reduce confounding factors. (4) The surgical procedure for all patients included in this study is sleeve gastrectomy, and no other surgical procedures are included.

## Conclusions

The depression levels in MBS patients are predominantly mild, showing a gradual declining trend within six months post-surgery. Anxiety is a risk factor for depression both preoperatively and within six months post-surgery. Patients with comorbid anxiety, low medication adherence, and high vitamin B12 levels exhibit higher preoperative depression severity and require particular attention. However, their postoperative depression levels decreased significantly, highlighting the importance of postoperative follow-up for effective emotional management in this subgroup.

## Data Availability

The raw data supporting the conclusions of this article will be made available by the authors, without undue reservation.
